# Skin dendritic cell and T cell activation associated with dengue shock syndrome

**DOI:** 10.1038/s41598-017-14640-1

**Published:** 2017-10-27

**Authors:** Huynh Thi Le Duyen, Daniela Cerny, Dinh The Trung, Jassia Pang, Sumathy Velumani, Ying Xiu Toh, Phan Tu Qui, Nguyen Van Hao, Cameron Simmons, Muzlifah Haniffa, Bridget Wills, Katja Fink

**Affiliations:** 1 Oxford University Clinical Research Unit, Hospital for Tropical Diseases, Ho Chi Minh City, Vietnam; 2 Singapore Immunology Network (SIgN), Agency for Science, Technology and Research (A*STAR), Biopolis, Singapore; 3 School of Biological Sciences, Nanyang Technological University, Singapore, Singapore; 4 Biological Resource Centre (BRC), Singapore, Singapore; 5 Hospital for Tropical Diseases, 764 Vo Van Kiet, Ho Chi Minh City, Vietnam; 6 University of Medicine and Pharmacy of Ho Chi Minh City, Ho Chi Minh City, Vietnam; 7 Department of Microbiology and Immunology, Peter Doherty Institute, University of Melbourne, Melbourne, Australia; 8 Institute of Cellular Medicine, Newcastle University, Newcastle upon Tyne, United Kingdom; 9 Centre for Tropical Medicine and Global Health, Nuffield Department of Clinical Medicine, University of Oxford, Oxford, United Kingdom

## Abstract

The pathogenesis of severe dengue remains unclear, particularly the mechanisms underlying the plasma leakage that results in hypovolaemic shock in a small proportion of individuals. Maximal leakage occurs several days after peak viraemia implicating immunological pathways. Skin is a highly vascular organ and also an important site of immune reactions with a high density of dendritic cells (DCs), macrophages and T cells. We obtained skin biopsies and contemporaneous blood samples from patients within 24 hours of onset of dengue shock syndrome (DSS), and from healthy controls. We analyzed cell subsets by flow cytometry, and soluble mediators and antibodies by ELISA; the percentage of migratory CD1a^+^ dermal DCs was significantly decreased in the DSS patients, and skin CD8^+^ T cells were activated, but there was no accumulation of dengue-specific antibodies. Inflammatory monocytic cells were not observed infiltrating the skin of DSS cases on whole-mount histology, although CD14^dim^ cells disappeared from blood.

## Introduction

Symptomatic dengue affects an estimated 100 million people worldwide each year^1^. Depending on factors such as age, pre-existing flavivirus immunity, and the dengue virus (DENV) serotype responsible for the current infection, 1–7% of symptomatic individuals develop severe disease^2^. Typically, this manifests with a vascular leakage syndrome characterized by hemoconcentration and serosal effusions, usually accompanied by thrombocytopenia and a coagulopathy^3–5^. Vascular leakage becomes clinically detectable around four to five days after fever onset, although it likely starts earlier but is initially compensated^6–8^. In severe cases, hypovolemic shock – i.e. dengue shock syndrome (DSS) – ensues, but fortunately in experienced hands the fatality rate of DSS can be as low as 0.1%^9^.

While vascular leakage is recognized as the pathognomonic feature of DSS, the underlying mechanisms contributing to the leakage, potential associations with immune cell activation, and the consequences for disease progression, are not well understood. Cellular aspects of severe dengue pathogenesis are difficult to study in humans due to limited access to tissue. Not much is known therefore about changes in cell composition and cell activation status that may contribute to leakage or other severe phenomena, or conversely, that may be affected by the DENV mediated vasculopathy. However, since human skin is a highly vascular organ that can be biopsied with relative ease, an opportunity exists to study blood vessels and tissue-resident immune cells alongside blood immune cells during acute infection. Human skin harbors several antigen-presenting cells (APCs) including dermal dendritic cells (DDCs) and epidermal Langerhans cells (LCs). DDCs comprise CD1a^+^ DDCs (also called CD1c^+^ DDCs^10^), and CD141^+^ DDCs, which have the capacity to cross-present antigen^11^. Dermal CD14^+^ cells fulfill DC-associated functions such as T cell activation, but are monocyte-derived and are genetically more related to macrophages than to dendritic cells^12^. Besides DDCs, skin also contains macrophages, which are non-migratory, in contrast to DCs^13^. In addition to these APCs that modulate immune responses during infection, inflammatory monocytes attracted by locally produced chemokines can infiltrate from blood vessels into the skin and contribute to inflammation at the site of infection, as shown in mouse models^10,14^. In humans CD14^+^ classical monocytes have the capacity to produce high amounts of cytokines after stimulation and are efficient phagocytes, while CD14^dim^CD16^+^ monocytes tend to “patrol” blood vessels slowly and then extravasate into tissues during inflammation^15^. In the context of infection, inflammatory monocyte-derived cells can be detrimental, for example if they infiltrate into the brain during encephalitic viral infection^16^. On the other hand, monocyte-derived cells can support virus clearance by contributing to T cell activation in the draining lymph node^17^. In dengue, monocyte-derived cells that infiltrate into the skin shortly after intradermal infection are a major infection target and likely contribute to the overall viral burden^10,14^.

In this study, we aimed to describe immune cell alterations in the skin of patients with significant DENV associated vascular leakage resulting in DSS, in order to gain insight into the tissue-associated pathology of severe dengue. Skin cells from DSS patients and healthy controls were analyzed by flow cytometry, and culture supernatants from skin cell preparations were assessed for the presence of cytokines and antibodies. We found evidence of immune cell activation in the skin of the DSS patients, notably a decrease the number of CD1a^+^ DDCs alongside the appearance of CD8^l^°^w^ T cells. In parallel, a decrease of CD14^+^ monocytes and a virtual loss of CD14^dim^ monocytes was observed in the blood, but there was no evidence that these cells infiltrated into interstitial spaces in the skin or increasingly adhered to blood vessels in the skin.

## Results

### DSS patients show a decrease in skin-resident CD1a^+^ DCs

17 young adults presenting with classical DSS were enrolled in the study (Table 1), together with 18 healthy university students that formed the control group. Dengue was confirmed by RT-PCR in 13/17 DSS cases and serologically in the remaining four patients; in all cases the serological responses was consistent with secondary infection. Following initial resuscitation and with written informed consent, shave biopsies were collected from the DSS patients a median (range) of 14 (4–20) hours after onset of shock. In all cases the biopsies were obtained from skin that appeared normal on visual inspection, with no rash or petechiae/bleeding evident. Minor bleeding occurred afterwards from the biopsy site in 5/17 DSS cases, with more significant oozing in one patient requiring application of a pressure bandage. All individuals recovered fully from shock with appropriate fluid resuscitation, including parenteral colloid therapy in 7/17 cases; no patient required blood products, inotropes or respiratory support. Skin biopsies and simultaneous blood samples were obtained from the healthy students in the outpatient department, without complications.

**Table 1:** Characteristics of patients involved in the study.

Characteristic	Dengue Shock Syndrome Cases (N = 17)
Age (years)	23 (15–33)
Male sex	9 (53)
BMI (kg/m^2^)	19.7 (16.4–26.4)
Illness day at enrolment (days)	6 (4–7)
Time from DSS onset to biopsy (hours)	14 (4–20)
Overall percentage hemoconcentration (%)	25 (11–46)
Minimum platelet count* (10^9^/L)	25 (11–54)
Required parenteral colloid therapy	7 (41)
Dengue diagnostics	
PCR Confirmed	13 (76.5)
Dengue IgM & IgG positive at presentation on day 6 (2 cases) or 7 (2 cases)	4 (23.5)
Dengue RT-PCR positive	13/17 (76)
Dengue 1	5
Dengue 2	3
Dengue 3	3
Dengue 4	2
Immune status	
Secondary infection	17 (100)

Data are presented as n (percentage) for categorical variables and median (range) for continuous variables.

*In several cases the platelet count dropped below 25 × 10^9^/L 1–2 days after the biopsy was taken.

**Figure 1:**
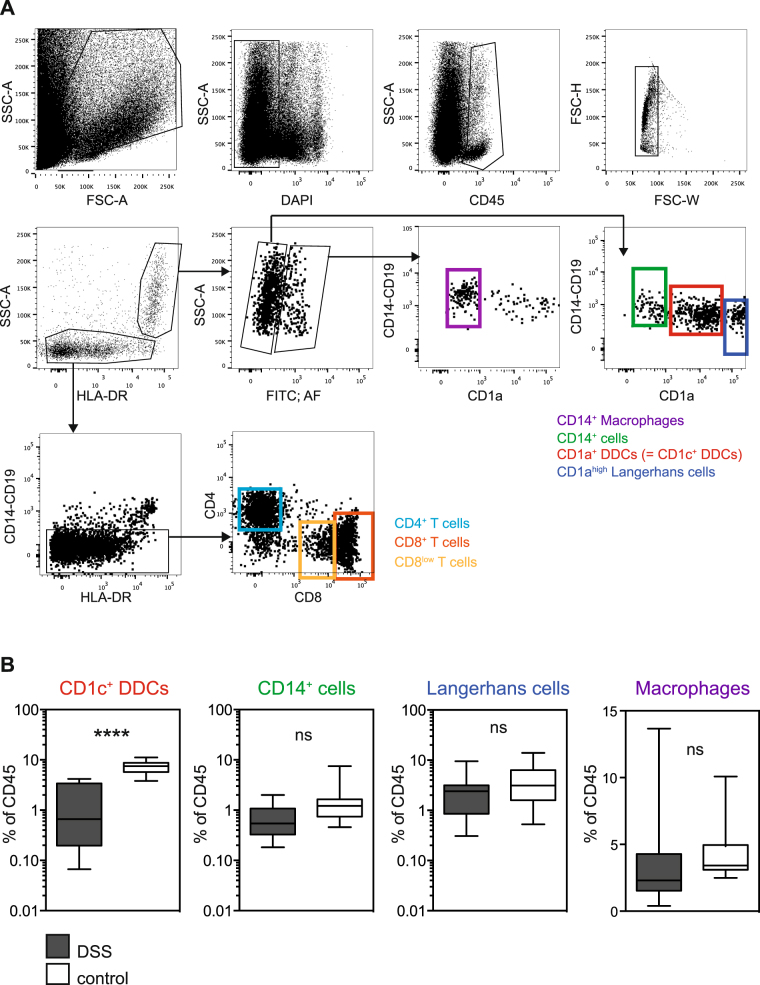
Flow cytometry analysis of skin biopsies from dengue patients shows evidence of APC activation. (**A**) Gating strategy used for the analysis of skin cells. (**B**) Distribution of CD1a^+^ DDCs, CD14^+^ cells, Langerhans cells and macrophages as gated in (**A**), compared between DSS cases (n = 15) and healthy controls (n = 18). Boxes and whiskers represent minimum, 25th percentile, median, 75th percentile, and maximum values. ****p < 0.0001. Unpaired t test.

Single cell suspensions were prepared from half of each fresh skin biopsy for flow cytometry analysis of DC subsets, macrophages and T cells (Fig. 1A), while the other half was fixed for histological analysis. Whole mount skin samples were stained with antibodies binding to endothelial cell marker CD31, lymphatic vessel cell marker Lyve-1, IgG and NS1. However, no obvious differences were observed between DSS cases and healthy controls.

Flow cytometry analysis of skin cells showed a significant, selective decrease of CD1a^+^ DDCs in DSS patients compared to healthy controls whereas the percentages of CD141^+^ DDCs, CD14^+^ cells and macrophages were comparable between the two groups (Fig. 1B). We also included antibody binding to CD19 in the panel, to detect potential accumulation of B cells, but no evidence of B cells was observed in either the DSS patients or the healthy controls.

**Figure 2:**
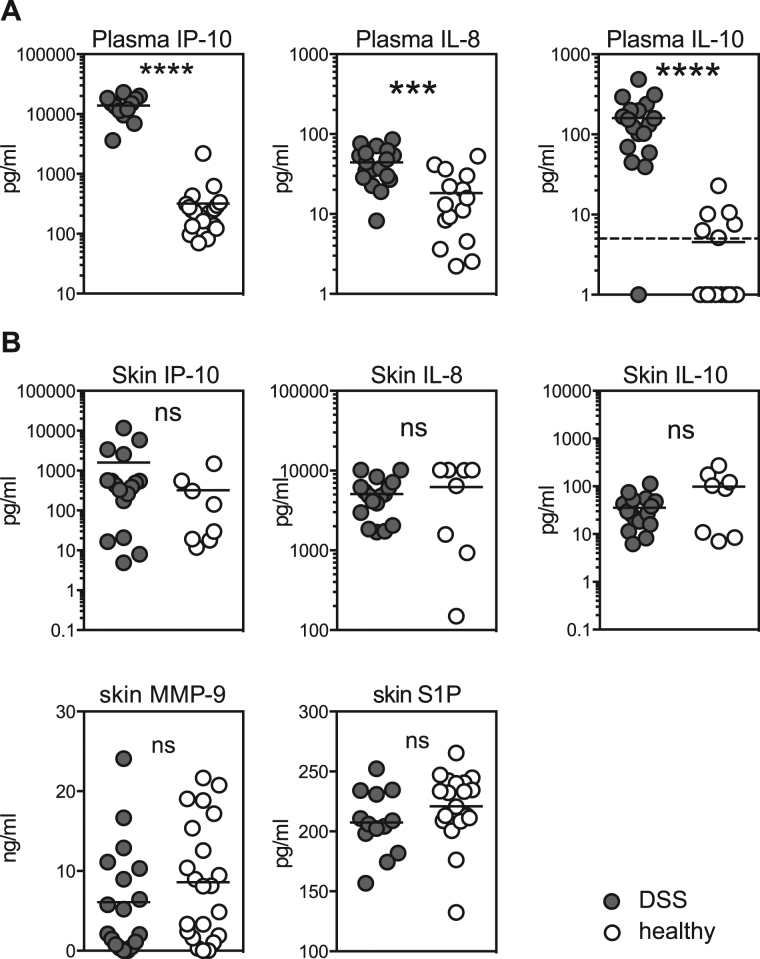
Cytokine levels in skin and blood. (**A**) Cytokines in the blood of DSS patients and healthy controls. The dotted line indicates the limit  of detection. (**B**) Cytokines in the supernatant of skin cell preparations from biopsies taken from DSS patients and healthy controls. Each dot represents one individual. Bars indicate means. ****p < 0.0001, ***p = 0.0004. Unpaired t test.

We investigated whether the apparent loss of the CD1a^+^ DDC population could have been triggered by inflammatory cytokines secreted by skin cells, comparing plasma values for a range of cytokines with levels measured in the medium in which the cells were digested overnight (see Methods section). Out of twelve cytokines analyzed, IP-10, IL-8 and IL-10 were significantly increased in the plasma of DSS patients but none of the cytokines analyzed were significantly increased in DSS skin (Fig. 2A,B). Specifically considering the vascular leakage-inducing soluble mediators MMP-9 and S1P, these were not increased in skin of DSS patients (Fig. 2B).

In summary, dermal DCs were activated in DSS patients during the critical phase for vascular leakage. However, no changes in monocyte-derived cells, dendritic cells or macrophages were observed, excluding infiltration into the skin of monocyte-derived cells from blood during acute dengue.

### T cells are significantly activated in the skin of dengue patients during shock

**Figure 3:**
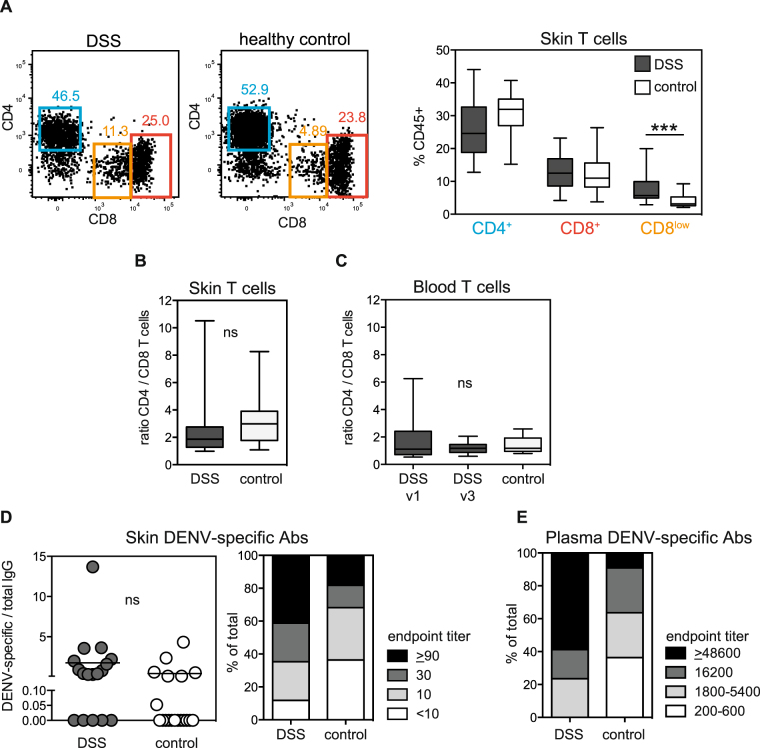
T cell activation and antibody analysis in the skin. (**A**) % of CD4^+^, CD8^+^ and CD8^low^ cells in the skin of DSS and control patients. ***p = 0.0003, Mann-Whitney test. Representative flow cytometry graphs are shown on the left. (**B**) CD4/CD8 T cell ratio in the skin. (**C**) CD4/CD8 T cell ratio in the blood. For DSS patients: v1 is during the acute phase when the skin biopsy was taken. v3 is an early convalescent time point. (**A**–**C**) Boxes and whiskers represent minimum, 25th percentile, median, 75th percentile, and maximum values. (**D**) Ratio of dengue-specific versus total IgG antibodies in the skin, analysed in the supernatants from skin cell preparations. Each symbol represents the mean concentration in the supernatant from one donor,. The distribution of different endpoint titers amongst DSS patients and healthy controls is shown in the right-hand graph. (**E**) For comparison with skin, the distribution of different endpoint titers amongst DSS patients and healthy controls in the blood is shown.

The principal function of CD1a^+^ DDCs is to activate T cells, either locally in the skin or after migrating to the draining lymph nodes. Given the apparent activation of these DDCs we tested whether skin T cells were activated as well (Fig. 1A). While the selected antibody panel did not include a specific activation marker due to the limited number of FACS detection channels, we observed a significant accumulation of T cells with down-regulated CD8 expression in DSS patients when compared to healthy controls (Fig. 3A), which we interpreted as a sign of activation^18^. For comparison, we also analysed T cells in the blood. A higher percentage of CD8^l^°^w^ cells was observed for blood T cells in DSS patients compared to healthy control but the difference was considerably less marked in blood (p = 0.02, data not shown) than in skin (p = 0.0003) (Fig. 3A). The CD4/CD8 T cell ratio, which can be decreased after viral infection, was not affected in the skin (Fig. 3B) or the blood of DSS cases compared to healthy controls (Fig. 3C).

### Dengue-specific antibodies do not accumulate in the skin in DSS

Immune complex formation during infection likely occurs in dengue patients, particularly during secondary infections, when a large amount of antibody is produced by re-activated dengue-specific memory B cells at a time when the concentration of viral antigens is high. The example of the Arthus reaction provides evidence that immune complexes adhering to skin blood vessels can contribute to vascular leakage^19^. To address whether DENV-specific Abs accumulate in the skin during DSS, concentrations of DENV-specific antibodies were measured by ELISA in the supernatants of single cell suspensions prepared from skin biopsies. To account for the different sizes of the skin biopsies, total antibody concentrations in the skin were also measured, and the ratio of DENV-specific/total IgG was used for comparison between samples. Overall, DSS patients had higher concentrations of dengue-specific IgG in the skin compared to healthy controls (Fig. 3D). However, a similar trend was observed in the blood (Fig. 3E). Increased titers of dengue-specific IgG in the blood after a recall response are expected. The increased IgG titers observed in DSS skin are therefore likely to reflect the systemic increase in dengue-specific IgG titers rather than skin-specific accumulation of these antibodies alone or in immune complexes.

### Systemic CD14^dim^ monocytes disappear from the blood of DSS patients but do not accumulate in the skin

**Figure 4:**
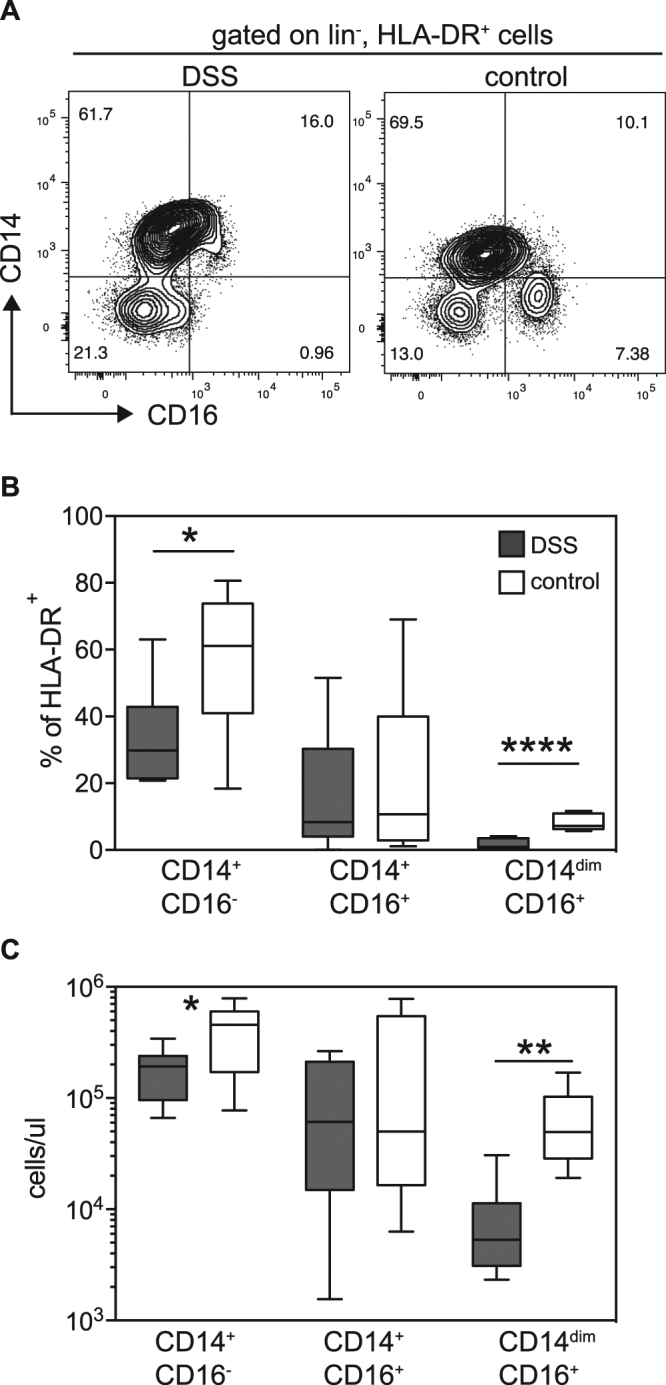
Monocyte analysis in whole blood of DSS patients and controls. (**A**) Representative flow cytometry plots for the analysis of CD16 and CD14 expression. Graphs were gated on HLA-DR^+^ cells. (**B**) Quantitative analysis of the flow cytometry data, comparing DSS with controls. *p = 0.033, ****p < 0.0001. Unpaired t test. (**C**) Absolute numbers of monocyte subsets shown in (**B**). *p = 0.033. Unpaired t test. **p = 0.0013. Mann-Whitney test.

Studies in IFNalpha/beta/gamma-receptor knock-out mice (AG129 mice) have shown that inflammatory monocytes infiltrate into the skin at the site of dengue infection around the time of peak viremia and that these cells are efficient amplifiers of infection^10,14^. In contrast, monocyte infiltration into the skin in areas unrelated to the injection site was much reduced but could still be observed in an adoptive transfer model of fluorescently labeled cells^14^. CD14^dim^ blood monocytes have the capacity to infiltrate into tissue and differentiate into tumor-necrosis factor (TNF)/inducible nitric oxide synthase (iNOS)-producing DCs (tipDCs). We analyzed the distribution of monocyte subsets in the blood of DSS patients during acute disease by flow cytometry (Fig. 4A). The percentages of CD14^+^ monocytes amongst HLA-DR^+^ cells, as well as absolute numbers of CD14^+^ monocytes, were decreased in DSS patients compared to healthy controls. More significant, however, was the disappearance of CD14^dim^ cells from the blood of DSS patients (Fig. 4B). We hypothesized that CD14^dim^ cells either adhered more to blood vessels or had migrated to tissues. We looked for evidence of these monocytes in the skin by histology but found CD16^+^ cells associated with blood vessels in only one out of 12 samples stained and analyzed (Supplementary Fig. 1). Furthermore, there was no significantly increased concentration of TNFα in the skin, providing additional evidence that tipDCs do not infiltrate into skin during severe dengue disease.

### Infiltration of inflammatory monocytes is restricted to the infection site as assessed in a non-human primate model

**Figure 5:**
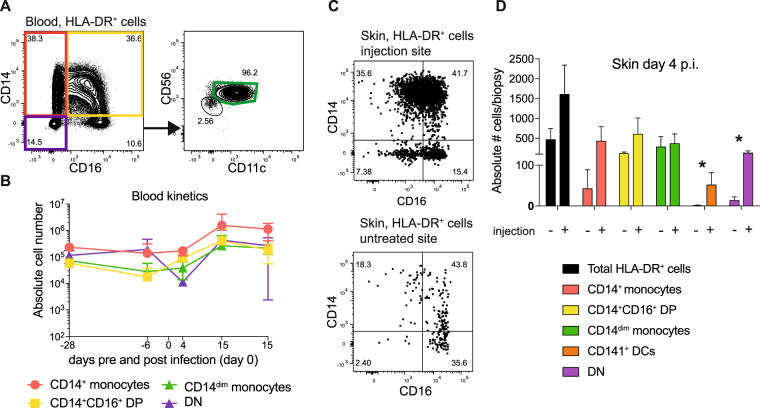
Cell infiltration at the infection site in cynomolgus macaques (CM). (**A**) Representative flow cytometry graphs showing the gating for monocytes in the blood of CM. (**B**) Quantitative analysis of the flow cytometry data as shown in (**A**), analyzed over time. Symbols are the means ± SD for n = 4. A repeated measures one-way ANOVA test showed no statistically significant changes. (**C**) Representative flow cytometry plots showing the gating for monocyte in the skin of CM at the injection site and a control site. (**D**) Quantitative analysis of the flow cytometry data, comparing injection site with control site. Bars are means ± SD, n = 4. Significant differences in absolute numbers of APCs per 8 mm-diameter punch biopsy were found for CD141^+^ DCs (p = 0.044) and for CD14^−^CD16^−^ DN cells (p = 0.025).

To compare cell infiltration at the site of virus inoculation with a control site we studied skin biopsies of cynomolgus macaques (CM) after intradermal infection. 2 × 10^5^ pfu DENV-2 was injected via the intradermal route in a 100 µl inoculum into the right medial thigh. All four infected CM used in this study developed viremia that peaked on day 1 (three animals) or day 3 (one animal), and cleared by day 7 after infection. Four days after infection 8 mm skin punch biopsies were taken from the injection site on the right thigh and from an uninvolved site on the left thigh. The skin samples were processed and analyzed by flow cytometry to study the composition of APCs (Fig. 5). In parallel, the blood monocyte composition was analyzed over time from 28 days before until 28 days after the infection. Unlike in the DSS patients, no decrease in CD14^dim^ monocytes was observed in CM on day 4 after infection, the time when viremia was decreasing or was already undetectable Fig. 5A). This difference in CD14^dim^ monocytes might be due to the much lower viremia in CM compared to human infections, and the lower generalised immune activation that occurs as a consequence. The CD14/CD16 double-negative population seemed to contract at day 4, but this was not statistically significant (Fig. 5B).

An obvious infiltration of monocytes into the skin was seen at the injection site when compared to the control site (Fig. 5C), in line with earlier results from AG129 mice^10,14^. We did not detect infection of infiltrating monocytes by flow cytometry, as was the case for AG129 mice. However, we did not have a positive CM cell infection control and could therefore not exclude a technical limitation. Significant differences in absolute numbers of APCs per punch biopsy were found for CD141^+^ DCs (p = 0.044) and for CD14^−^CD16^−^ DN cells when comparing injection site and control site (p = 0.025). DN cells comprised of HLA-DR^+^ cells that were CD56^+^, CD206^−^, CD11c^l^°^w^ and CD141^−^ (Supplementary Fig. 2).

In both the mouse and the CM model, DENV infection caused accumulation of monocyte-derived cells at the site of infection in the skin. However, cell infiltration was not apparent elsewhere in the skin, in line with the observations in human patients. While it is difficult to compare the timing of vascular leakage in DSS patients with animal models of dengue infection, we made the assumption that viremia is the key factor triggering cellular manifestations and the time window of our investigations was during peak viremia for mice and during the tail end of viremia for both CM and patients.

## Discussion

The pathogenesis of severe dengue remains unclear, in particular the mechanisms underlying the increase in vascular permeability that is the hallmark of severe disease. No specific structural abnormalities have been detected in the microvasculature of dengue-infected patients but biopsy studies to date have been limited and have primarily focused on investigating the skin rash^20–22^. Instead, a cascade of immunological mediators generated in response to the infection are thought to alter microvascular function in some way, but debate continues at to which immune cells and/or mediators are primarily involved, and how these factors interact with the microvasculature.

In this study, we addressed the question whether myeloid cell activation and mobilisation was apparent in the skin during acute dengue, and whether this could be a potential driver or amplifier of vascular pathology. The selective decrease in CD1a^+^ DDCs that we observed could be a result of high systemic levels of inflammatory cytokines measured in the blood of DSS patients. For example, it has been found that *in vitro* incubation with IL-10 predominantly promotes migration of CD1a^+^ DDCs from healthy donor skin explants^23^. Antigen-presenting CD1a^+^ DDCs are mobilized and migrate to draining lymph nodes to activate specific T cells travelling through these lymph nodes. We speculate that the presentation of DENV antigen in skin draining lymph nodes during infection contributes to the activation of dengue-specific T cells and their migration to the skin. An accumulation of dengue-specific T cells has previously been observed in skin blisters raised in patients during acute dengue infection^24^. The activated CD8^low^ T cells in the skin that we detected in this study might be part of those cells recruited upon infection. Alternatively, given that all subjects were secondary dengue cases, the CD8^low^ T cells could represent activated DENV-specific tissue-resident T_RM_ cells from the previous infection, which were already resident in the skin at the time point of infection^25^.

Besides the mobilization of CD1a^+^ DDCs in the skin we found perturbations of myeloid cells in the blood, in particular a significant loss of CD14^dim^ cells, and a decrease in CD14^+^ inflammatory monocytes. CD14^dim^ cells patrol vessels and secrete TNFα, IL-1b and CCL3 in response to viral infection^15^. Given their patrolling behavior we considered whether increased adherence of CD14^dim^ cells to microvessels in dengue patients could explain their disappearance from blood. However, at least in the skin no evidence of increased numbers of HLA-DR^+^ cells was observed by flow cytometry (Fig. 1) and no evidence of CD16^+^ cells was observed in histology (S. Fig. 1). This does not exclude accumulation of CD14^dim^ cells in other organs. Similar to our findings, another study that analyzed blood samples from a Sri Lankan cohort of dengue fever and DHF cases found a decrease in CD14^+^ and CD14^dim^ monocytes, in line with our findings. However, that report focused on an increase in CD14^+^CD16^+^ monocytes in blood, which was not observed in our study^26^. The different findings could be due to different processing and staining protocols, possibly the use of whole blood versus PBMCs.

One important limitation to note is that for practical reasons we were unable to recruit a control group of dengue-infected individuals without vascular leakage at the same time-point in the evolution of the illness. In DSS cases the presence of significant leakage can be identified at the onset of shock, while in less severely affected individuals determination of the presence or absence of leakage is typically made retrospectively when hemo-concentration resolves^27,28^. In order to capture a patient group without leakage, biopsies would have had to be taken in a large number of individuals, many of whom would later be identified to have some degree of leakage and/or would have proved negative for DENV infection. With the current data therefore, we are unable to say whether the findings are representative of dengue infection in general rather than being characteristic of dengue-associated vasculopathy. Future studies could be designed to include other patient groups to explore potential correlations with severity. We are also not able to comment on whether our findings are specific to dengue infection. We did attempt to enrol non-dengue disease controls, admitted to ICU with illnesses of similar severity but caused by other pathogens (e.g. viral encephalitis, tetanus). However, very few families gave consent for a biopsy to be performed and this control group had to be excluded from the analysis.

The use of skin to address questions regarding the vasculopathy may also be questioned, since clinical leakage is most obvious from serosal surfaces (pleural effusions, ascites), while skin edema typically becomes apparent only after fluid resuscitation in DSS cases. However, it is clear that complex hydrostatic/oncotic pressure relationships determine the relative prominence of leakage from serosal surfaces versus other microvessels^29,30^ and that the severity of hypoproteinemia seen in DSS can only be explained by a generalized pathophysiological process involving the microvasculature in all organs^4,27^. A previous study by Saadiah *et al*. reported the histological analysis of skin biopsies from 32 patients with dengue haemorrhagic fever (DHF), 6 of whom subsequently developed DSS^31^. The aim of that study was to assess histology of skin biopsies as a potential prognostic tool to predict severe disease. The biopsies were stained with antibodies specific for CD3, CD68, CD1a, VEGF, CD25 and TNFα. The authors described that “almost invariably mild” abnormalities consisting of minor inflammatory infiltrates, mostly histiocytes (macrophages and/or dendritic cells) and T cells, were observed in all cases. Only six out of 32 DHF patients had more pronounced histiocyte infiltration, but there was no correlation with final disease severity.

In conclusion, in this novel study skin biopsies obtained from Vietnamese young adults with hypovolemic shock secondary to dengue-associated vasculopathy showed little direct evidence of vascular or immune-related pathology. However, there was evidence of immune cell activation consistent with a role for skin as an immune organ that induces T cell responses in dengue. While further studies are required to address the protective capacity of skin-associated T cells, vaccination via the intra-dermal route should be considered as alternative to the subcutaneous or intra-muscular routes of immunization^32,33^. The absence of cellular infiltration, soluble mediators, and antibodies in the skin, despite profound disturbances in permeability, suggests that alternative mechanisms need to be explored. Recently dengue non-structural protein 1, a soluble protein secreted by the virus during replication and often present in high concentrations during the acute phase, has been suggested to have a role in dengue-associated microvascular dysfunction, potentially through interactions with constituents of the endothelial surface glycocalyx^34,35^. This layer, which lines all blood vessels and is thought to be the primary barrier regulating intrinsic vascular permeability, is singularly difficult to examine in human tissues but merits further interrogation.

## Material and Methods

### Clinical Methods

The study was approved by the Ethical Committee of the Hospital for Tropical Diseases of Ho Chi Minh City, and the Oxford Tropical Research Ethics Committee. All experiments were performed in accordance with guidelines and regulations stipulated by the approving committees.

Young adults (15 years or older) admitted to the Intensive Care Unit at the Hospital for Tropical Diseases in Ho Chi Minh City with established DSS (pulse pressure ≤20 mmHg or hypotension for age, with tachycardia and/or signs of impaired peripheral perfusion) were eligible for recruitment to the study provided they were approached after cardiovascular stabilization, had no mucosal or significant skin bleeding, and the platelet count was at least 25,000 mm^3^. An experienced ICU physician discussed the study with potential participants after the initial resuscitation, and if the patient or parent/guardian gave written informed consent (and minors aged 15–17 years provided assent) the subject was enroled.

Within 24 hours of DSS onset a shave biopsy of approximately 5 × 5 mm was taken from the posterior iliac crest by the same physician, under local lignocaine anaesthesia using DermaBlades® (Personna Medical). Any immediate bleeding was controlled using a Model 2000 hyphrecator (ConMed), and the site was observed regularly for 48 hours. An EDTA research blood sample was obtained simultaneously. All participants were reviewed daily by the study physician, using a simple daily case report form, and additional research blood samples were obtained at discharge and follow-up one month later. Comprehensive dengue diagnostics were carried out on paired samples, including RT-PCR and in-house Capture IgM and IgG ELISAs, following established methodology^36–38^. The DSS patients were considered to have confirmed dengue if the RT-PCR was positive or if the IgM and IgG responses were positive in patients presenting after illness day 5. The infection was classified as secondary if the IgM/IgG ratio was greater than 1.8 in the second week of illness.

To enroll healthy control participants, flyers were distributed at a local university describing the study. Interested students were asked to attend an initial individual assessment, at which those who were generally healthy and with no history of febrile illness for 3 months were given more detailed information; 18 individuals (median (range) age 23 (22–26) years, 50% male) who were happy to proceed gave written consent and were invited to return another day for the biopsy and blood sampling.

Biopsies were placed immediately in a container with RPMI medium. For the preparation of single cell suspensions the biopsy was cut into small pieces and digested overnight at 37 °C in RPMI medium containing 0.8 mg/ml Collagenase (Type IV, Worthington-Biochemical) and 100 U/ml DNase (Roche). Connective tissue and debris were removed by filtering the cells through a piece of 70 μm filter fabric.

### Whole mount histology of skin biopsies

The part of the fresh skin biopsy that was not used for flow cytometry was fixed in 2%PFA/30% sucrose at 4 °C overnight. The fixed piece were washed in 30% sucrose in PBS for 2 hours and stored in PBS at 4 °C until use. After removing fat tissue with scissors, biopsies were incubated with mouse anti-human CD16-FITC and rabbit anti-human CD31 overnight, followed by incubation with goat anti-rabbit AF568. After washing in PBS, skin pieces were mounted in ProlongGold, using a slide and coverslip to immobilize the pieces as flat as possible for imaging. Images were taken on an inverted Olympus IX81 microscope.

### Flow cytometry

Skin single cell suspensions were centrifuged at 500 g for 5 min after overnight incubation with collagenase and DNAse. The medium was stored at −20 °C for later antibody and cytokine analysis. The cell pellets were re-suspended in FACS buffer containing the antibodies for surface staining as listed in Table 2. Cells were washed once and re-suspended in FACS buffer containing 0.5 ug/ml DAPI for acquisition. For staining of peripheral blood cells, 200 microliters of blood collected in EDTA tubes was incubated with antibodies for surface staining as listed in Table 2. After 30 min incubation, red blood cells were lysed using Pharmlyse buffer (Becton Dickinson). Blood cells were re-suspended in FACS buffer containing 1% formalin for analysis on a FACSCanto (Becton Dickinson BD).

**Table 2:** Antibodies used in the study.

**Fluorescent dye**	**Marker**	**Clone**	**Marker**	**Clone**
	***Human blood***		***Human skin***	
PercP-Cy5.5	CD8^**a**^	SK1	CD8	SK1
FITC	CD3^**a**^	UCHT1	Autofluorescence	
PE	CD19^**a**^ NKp46^**a**^	HIB19 9E2	CD14	M5E2
PE-Cy7	HLA-DR^**b**^	L243	HLA-DR	L243
V500	CD16^**a**^	3G8	CD45	HI30
V450	CD14^**a**^	M5E2		
APC-Cy7	CD4^**a**^	RPA-T4	CD4	RPA-T4
APC			CD1a	HI149
UV Blue	DAPI^c^	NA	DAPI^b^	NA
	***CM blood***		***CM skin***	
FITC	CD1a^d^	NA1/34-HLK	CD1a	NA1/34-HLK
PerCP	HLA-DR^**b**^	L243	HLA-DR	L243
PE-Texas Red	CD14^f^	RMO52	CD14	RMO52
PE-Cy7	CD11c^a^	3.9	CD11c	3.9
BV421 (PB)	CD206^a^	19.2	CD206	19.2
PE	CD141^a^	1A4	CD141	1A4
Biotin (use with Strep-BV510)	CD1c^e^	AD5–8E7	CD1c	AD5-8E7
BV605	CD123^a^	7G3	CD123	7G3
APC-Cy7	CD16^b^	3G8	CD16	3G8
APC	E protein (intracellular)	4G2	E protein (intracellular)	4G2
BUV395	CD56^a^	NCAM16.2	CD56	NCAM16.2
UV Blue^+^	LIVE/DEAD Fixable^g^	NA	LIVE/DEAD Fixable	NA

^a^BD Biosciences, ^b^Biolegend, ^c^Sigma, ^d^Miltenyi, ^e^Abcam, ^f^Beckman Coulter, ^g^Molecular Probes.

### Antibody detection by ELISA

Total IgG antibodies were quantified using goat anti-human Ig coating antibody (H17000, Caltag Invitrogen), followed by incubation of diluted serum or skin cell supernatant. IgG was detected using goat anti-human IgG-HRP antibody (Sigma A0170). A human IgG standard was included at a range of 200–0.2 ng/ml.

Dengue-specific IgG was detected by coating ELISA plates with UV-inactivated, PEG-precipitated DENV particles, followed by incubation with plasma or skin cell supernatant and detection with goat anti-human IgG-HRP. For both ELISAs 5,5′-Tetramethylbenzidine (TMB, from Sigma) was used for color development. The color reaction was stopped with 1 M HCl before measurement of OD450.

### Cytokine measurements

The following cytokines were measured with Luminex technology and a 12-plex human cytokine panel (Millipore): IL-10, IL-1ra, IL-1a, IL-6, IL-8, IFN-g, IP-10, MCP-1 = CCL2, TNFa, VEGF, Fractalkine, MDC = CCL22. These cytokines were chosen based on a preliminary analysis with the 37-plex human inflammation panel and IL-21, IL-23 and IL-27 in a separate panel (Millipore). Most of the cytokines were either not detected or were equally present in healthy donors and patients, and therefore were not included the second analysis. S1P and MMP-9 in skin supernatant was quantified with commercial ELSIA kits (Catalog number E1860Hu-96 from Biotrend and Catalog number QIA56-1EACN from Merck).

### NHP experiments

Four purpose-bred, 3–5.5 year old male *Macaca fascicularis* were used in this study. The animals were supplied by KHI Bioservices Limited (Nafovanny), Vietnam, and were housed in the Biological Resource Centre BRC, Singapore. The experiments were reviewed and approved by the Institutional Animal Care and Use Committee, IACUC # 151008.

At day 0, animals were sedated and then immediately weighed and their body temperature recorded. A pre-dosing blood sample was taken from the left femoral vein, followed by intradermal administration of 100 µl of the DENV strain D2Y98P into the right medial thigh, using a 30 G tuberculin syringe. At day 4 after infection, 2 skin biopsies were performed using an 8 mm biopsy punch at the site of inoculation as well as another site on the left medial thigh as a control. The skin was closed using 3–0 monocryl simple interrupted or continuous intradermal sutures, and secured with vetbond. All skin samples were placed in 5 ml of media in a 15 ml falcon tube and kept on ice until processing. 0.3 mg/kg Buprenorphine was administered *i.m*. prior to recovery from anaesthesia.

Skin biopsies were processed as follows. Fat tissue and the lower dermis, which does not contain any immune cells, were removed with scissors. The remaining skin was cut into small (approx. 2 × 2 mm) pieces and incubated in collagenase and DNAse as described for the human skin biopsies. Cells were centrifuged and resuspended in FACS buffer containing the surface stain (Table 2). Cells were then fixed in 2% PFA, permeabilized and stained intra-cellularly with 4G2 (anti-DENV E protein) antibody. A FACS LSRII (Becton Dickinson) was used for acquisition of the samples.

## Electronic supplementary material

Supplementary Figures
