# Supplementary material for: Skin dendritic cell and T cell activation associated with dengue shock syndrome
Source: Sci Rep. 2017 Oct 27;7:14224. doi: 10.1038/s41598-017-14640-1 (PMC5660158; doi:10.1038/s41598-017-14640-1)
Supplement: Supplementary file 1 — Supplementary Figures [file 41598_2017_14640_MOESM1_ESM.pdf]

Supplementary Information for the manuscript:

Skin dendritic cell and T cell activation associated with dengue shock syndrome

Huynh Thi Le Duyen[1], Daniela Cerny[2,3], Dinh The Trung[1], Jassia Pang[4], Sumathy Velumani[2], Ying Xiu Toh[2], Phan Tu Qui[5], Nguyen Van Hao[5,6], Cameron Simmons[1,7], Muzlifah Haniffa[8], Bridget Wills[1,9*], Katja Fink[2,3*]

Supplementary Figure 1:

No consistent accumulation of CD14^{dim}CD16^{+} cells in the skin of DSS patients. Skin biopsies from DSS patients and healthy controls stained for endothelial cell marker CD31 (red) and monocyte marker CD16 (green). CD16^{+} cells were observed for patient . Numbers on the left of the image series indicate the length/heights of the square pictures. HC: healthy control, DSS: Dengue shock syndrome

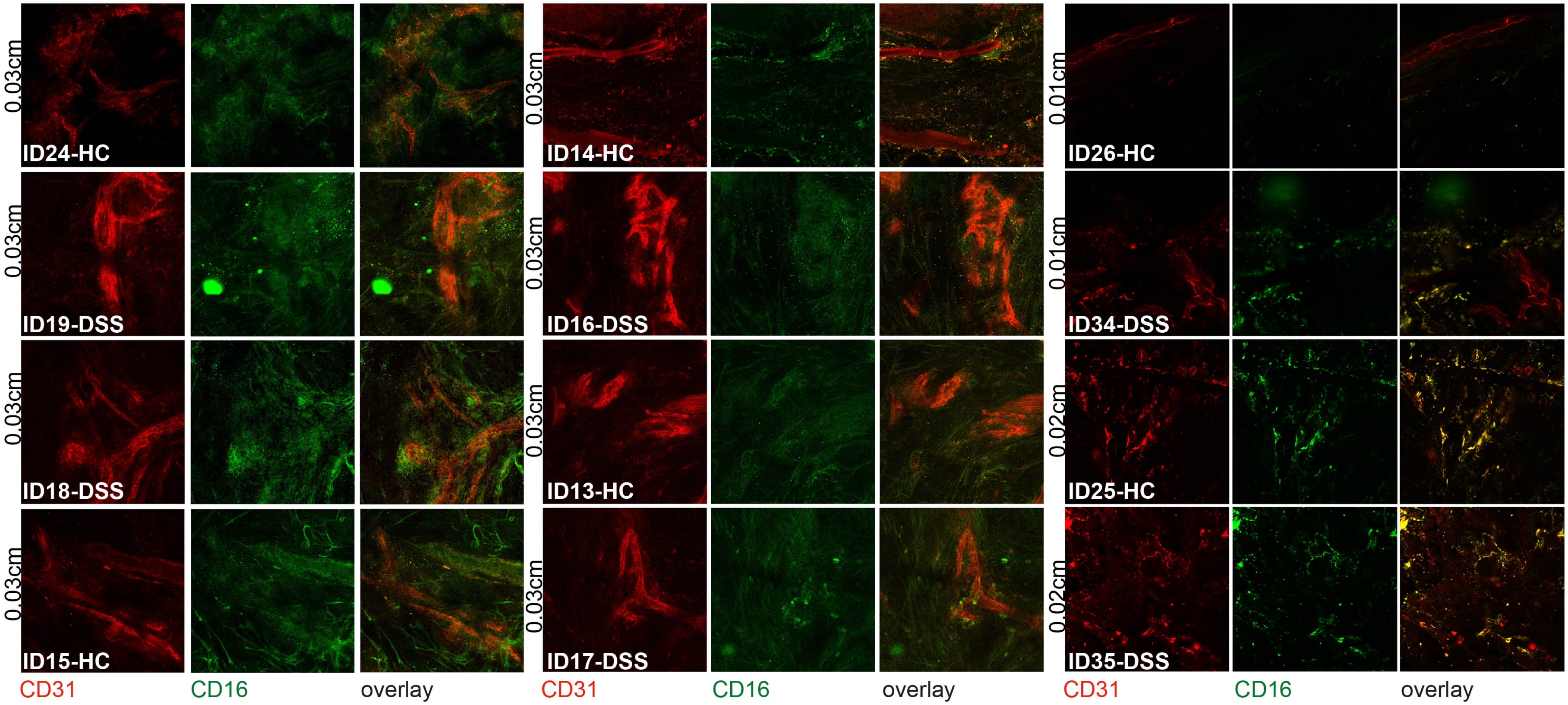

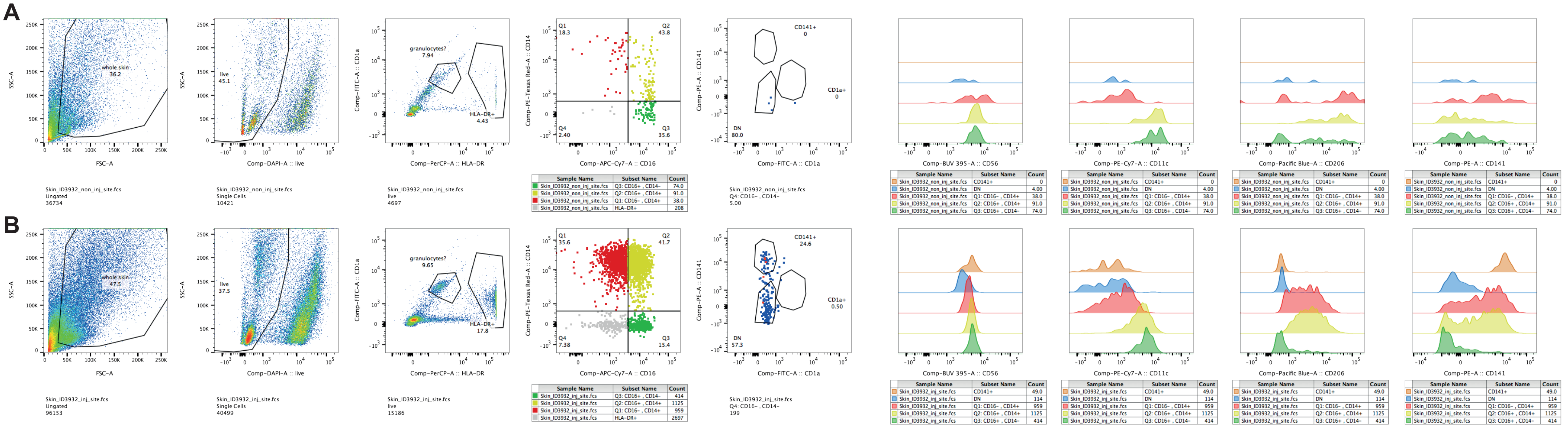

Supplemenatry Figure 2: Gating strategy for CM skin cells and expression levels of CD56, CD11c, CD206 and CD14 on monocyte and dendritic cell subsets. A) Skin cells from the untreated control site. B) Skin cells from the injection site. Skin biopsies were collected four days after infection.
